# Editorial: Evidence on the benefits of integrating mental health and HIV into packages of essential services and care

**DOI:** 10.3389/frph.2024.1454453

**Published:** 2024-07-12

**Authors:** Olumide Abiodun, Theddeus Iheanacho, Saheed Akinmayowa Lawal

**Affiliations:** ^1^ Department of Community Medicine, Babcock University, Ilishan-Remo, Ogun State, Nigeria; ^2^ Yale Institute of Global Health, Yale University, New Haven, CT, United States; ^3^ Department of Public Health, Babcock University, Ilishan-Remo, Ogun State, Nigeria

**Keywords:** mental health, HIV, service integration, health outcome, quality of life

**Editorial on the Research Topic** Evidence on the benefits of integrating mental health and hiv into packages of essential services and care

## Introduction

People living with HIV (PLWH) suffer disproportionately higher levels of mental disorders than the general population, both in high-income and low- and middle-income countries (1). There is also evidence to suggest that the burden of mental disorders is worse `in HIV compared to other chronic diseases (2). Mental disorders negatively affect client engagement and care retention and result in significant distortions in health outcomes at every stage of the HIV care continuum (1). The need for mental health care for PLWH is critical to mitigate HIV transmission and progression and improve clinical outcomes by creating awareness for mental disorders and integrating mental health services into the HIV care continuum. The Joint United Nations Programme on HIV/AIDS (UNAIDS) and World Health Organization (WHO) have underscored the importance of integrating HIV and mental health services, considering that both conditions accentuate each other's risk. Also, Integrated HIV-mental health approaches lead to better health outcomes, overall well-being and quality of life (3). More so, the “feasibility and acceptability of integrating mental health screening into an existing community-based program for prevention of mother-to-child transmission of HIV targeted at pregnant women and their male partners” is acceptable (4). For success, service integration should conform with all the critical elements of integrated service delivery as outlined by the WHO- “*the management and delivery of health care services so that the clients receive a continuum of preventive and curative services that cater to their needs over time and across different levels of the health system”* (5).

This editorial highlights some of the benefits of HIV-mental health services integration by introducing nine manuscripts published as a collection in response to the Research Topic: *Evidence on the Benefits of Integrating Mental Health and HIV into Packages of Essential Services and Care*. The manuscripts are from both high-income and low- and middle-income countries. Specifically, three are from China, two from the United States of America and one each from Ethiopia, Guinea, Kenya and Nigeria. The manuscripts (1) underscore the high prevalence of mental disorders among people living with HIV, (2) demonstrate that mental disorders lead to suboptimal health outcomes among PLWH, and (3) demonstrate the value of integrating mental health care into HIV care programs (Table 1).

**Table 1:** Summary of studies in this collection.

s/n	Study theme	Title	Authors	Setting Study	Study Design	Objective(s)	Sample size	Study population
1	HIV infection is disproportionately associated with mental disorders	Depression and Perceived stress among perinatal women living with HIV in Nigeria	Akinsolu et al	Southwest, Nigeria	Cross-sectional	To determine the prevalence and factors associated with depression and psychological stress	402	Pregnant or recently delivered women (within two years) living with HIV aged 19 to 49 years
2		Association between depression and HIV infection vulnerable populations in United States adults: a cross-sectional analysis of NHANES from 1999 to 2018	Xu et al	United States of America	Cross-sectional	To review and evaluate the association between depression and HIV infection	16,584	HIV adult vulnerable populations
3		Loneliness as a mediation from social support leading to a decrease of health-related quality of life among PLWHIV	Qian et al	China	Cross-sectional	To investigate the potential mediation mechanism of loneliness between social support and HRQoL	201	Adults accessing HIV care in a hospital
4		HIV-related stress predicts depression over five years among people living with HIV.	Liu et al	China	Longitudinal observational	To explore the longitudinal relationship between HIV-related stress, social support, and depression among people living with HIV	320	Adults living with HIV
5		Prevalence of suicide ideation among HIV/AIDS patients in China: A systematic review and meta-analysis	Li et al.	China	Systematic Review	To comprehensively analyze the prevalence of suicidal ideation among HIV/AIDS patients	6,174	Cross-sectional studies of adults with a sample size greater than 25
6		Implementation of trauma-informed care and trauma-responsive services in clinical settings: a latent class regression analysis	Anderson et al	Southeastern United States of America	Cross-sectional	To identify subgroups of HIV clinics based on their unique profiles of inner setting characteristics and assess how subgroup membership is related to the degree of TIC implementation and number of trauma-responsive services offered	317 (and 47 clinics)	Employees of HIV clinics
7	Mental disorders adversely affect HIV-related health outcomes	Loneliness as a mediation from social support leading to a decrease of health-related quality of life among PLWHIV	Qian et al.	China	Cross-sectional	To investigate the potential mediation mechanism of loneliness between social support and HRQoL	201	Adults accessing HIV care in a hospital
		Prevalence of suicide ideation among HIV/AIDS patients in China: A systematic review and meta-analysis	Li et al.	China	Systematic Review	To comprehensively analyze the prevalence of suicidal ideation among HIV/AIDS patients	6,174	Cross-sectional studies of adults with a sample size greater than 25
		Factors influencing self-efficacy for self-management among adult people with human immune deficiency virus on antiretroviral therapy in public hospitals of south-west Ethiopia	Aldisa et al	Southwest, Ethiopia	Cross-sectional	To identify factors influencing self-efficacy for HIV self-management	413	Adults accessing antiretroviral therapy in public hospitals
8	HIV-mental health service integration is beneficial	Exploring experiences of HIV care to optimize patient-centred care in Conakry, Guinea: a qualitative study	Kolie et al	Conakry, Guinea	Qualitative exploratory	To describe the patient-provider relationship and explore the challenges to optimal and patient-centred care	17 in-depth interviews and six focused group discussions	Adults accessing HIV care and caregivers in urban health facilities
9		Higher rates of mental health screening of adolescents recorded after provider training using simulated patients in a Kenyan HIV clinic: results of a pilot study	Concepcion et al	Thika, Kenya	Qualitative exploratory and interrupted time series	To design and pilot an evidence-based provider training strategy, simulated patient encounters	1,154	Adolescent girls and young women seeking health services at public HIV clinics
		Implementation of trauma-informed care and trauma-responsive services in clinical settings: a latent class regression analysis	Anderson et al	Southeastern United States of America	Cross-sectional	To identify subgroups of HIV clinics based on their unique profiles of inner setting characteristics and assess how subgroup membership is related to the degree of TIC implementation and number of trauma-responsive services offered	317 (and 47 clinics)	Employees of HIV clinics

## HIV infection is disproportionately associated with mental disorders

PLWH experience disproportionately high levels of many common mental disorders. Depression is significantly associated with HIV infection in studies from Nigeria, the US and China (Akinsolu et al., Xu et al., Qian et al., Liu et al.). The accentuated burden of depression in HIV, which likely exceeds that of other chronic diseases, is thought to be mediated by HIV-related stress (2). Depression is also commoner among HIV-vulnerable populations (Xu et al.), suggesting that the drives of HIV infection may also drive depression. This collection emphasizes the mediatory role of stress and loneliness in HIV-related depression. Stress is prevalent among PLWH (Akinsolu et al.), and stress and loneliness are potent predictors of depression and anxiety among PLWH, especially in the early stages of HIV infection (Liu et al., Qian et al.). Also, suicidal ideation is prevalent and rising among PLWH (6, 7). A meta-analysis of sixteen Chinese studies shows that about one-third of PLWH had suicidal ideation (Li et al.). Furthermore, interpersonal violence is also common among PLWH (Anderson et al.).

Although it has been demonstrated that mental disorders are commoner among PLWH than the general population, it is unlikely that all HIV subpopulations are equally vulnerable to mental disorders. This collection shows that men, homosexuals, unmarried and the depressed are more affected by suicidal ideations. Also, longer periods since HIV diagnosis and lower CD4 cell counts were associated with a higher risk of suicidal ideation (Li et al.). Other factors that are related to mental disorders in PLWH include being female, serodiscordant partners, low-income levels, lack of family support, duration on ART and the gestational age among HIV-positive pregnant women (Akinsolu et al.).

## Mental disorders adversely affect HIV-related health outcomes

Mental disorders are associated with HIV progression, poor medication adherence and exacerbation of the social and economic barriers to accessing HIV care, resulting in poor health outcomes and suboptimal quality of life (8). In this collection, Qian et al. used a Structural Equation Model to demonstrate a link between loneliness and reduction in health-related quality of life. Suicidal ideation is also associated with lower CD4 counts (Li et al.), while low self-efficacy is also related to drug side effects (Abdisa et al.). More so, the social context and stigmatising social process in which PLWH live, causes them to be stigmatised which further affects HIV-related health outcomes (9).

## HIV-mental health service integration is beneficial

Integrating mental health services into HIV care programs has the potential to mitigate the risk of disease progression and engender better health outcomes. Integrated service delivery also increases health system efficiency and patient satisfaction. Although available evidence demonstrates the value of service integration, health system challenges, including human resources, infrastructure and supply chain management, often constitute significant hindrances. Critical success factors include human capital development, awareness creation, stakeholder ownership and commitment and continuous health system development (5).

This collection also demonstrates the benefit (real or potential) of implementing mental health care interventions within HIV care programs. Integration of psychosocial counselling into HIV care promotes confidentiality, provider availability and care, improved access to antiretrovirals and patient preferences. These factors in turn optimize patient-centred care and result in better health outcomes for people living with HIV (Kolie et al.). Also, building the capacity of HIV caregivers will increase the enhance the diagnosis and referral of mental disorders. Concepcion et al. piloted a three-day Simulated Patient Encounter training on HIV care providers in Kenya. The study shows that evidence-based provider training can improve their competencies and service delivery for common mental disorders in HIV care settings. Anderson et al. underlined the significance of implementing trauma-informed care and trauma-responsive services in HIV settings to avoid re-traumatization in those with experience of intimate partner violence. The study also demonstrates that the success of HIV-mental health integration strategies hinges on the appropriate characterization of health facilities based on critical success factors depending on the core issue under consideration.

In summary, mental disorders disproportionately affect PLWH and result in poor HIV and mental health outcomes. Integrating mental health and HIV into Packages of Essential Services and Care will help recognize and address mental health needs and result in better health outcomes among PLWH.
